# Ethyl 4-(2,4-difluoro­phen­yl)-6-methyl-2-oxo-1,2,3,4-tetra­hydro­pyrimidine-5-carboxyl­ate

**DOI:** 10.1107/S1600536809015918

**Published:** 2009-05-07

**Authors:** Hoong-Kun Fun, Chin Sing Yeap, M. Babu, B. Kalluraya

**Affiliations:** a X-ray Crystallography Unit, School of Physics, Universiti Sains Malaysia, 11800 USM, Penang, Malaysia; b Department of Studies in Chemistry, Mangalore University, Mangalagangotri, Mangalore 574 199, India

## Abstract

In the title compound, C_14_H_14_F_2_N_2_O_3_, the dihydro­pyrimidin­one ring adopts a flattened boat conformation. The difluoro­phenyl group is disordered over two orientations with occupancies of 0.544 (3) and 0.456 (3). The methoxy­carbonyl group is disordered over two positions with occupancies of 0.580 (8) and 0.420 (8). In the crystal, mol­ecules are linked into centrosymmetric dimers by paired N—H⋯O hydrogen bonds and the dimers are linked into a ribbon-like structure along [100] by further N—H⋯O hydrogen bonds.

## Related literature

For general background and pharmaceutical applications of pyrimidinones, see: Kalluraya & Rai (2003 ▶); Atwal (1990 ▶); Sadanandam *et al.* (1992 ▶); Steele *et al.* (1998 ▶); Manjula *et al.* (2004 ▶). For bond-length data, see: Allen *et al.* (1987 ▶). For the stability of the temperature controller used for the data collection, see: Cosier & Glazer (1986 ▶).
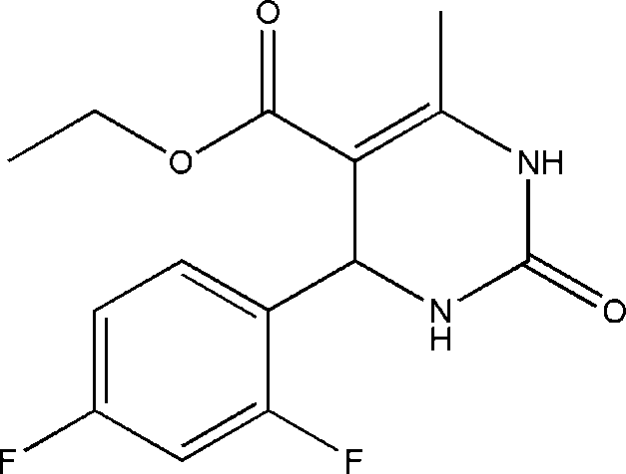


## Experimental

### 

#### Crystal data

C_14_H_14_F_2_N_2_O_3_
*M* *_r_* = 296.27
Triclinic, 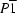

*a* = 7.5176 (1) Å
*b* = 8.0483 (1) Å
*c* = 11.9323 (2) Å
α = 90.147 (1)°
β = 100.839 (1)°
γ = 108.421 (1)°
*V* = 671.25 (2) Å^3^
*Z* = 2
Mo *K*α radiation
μ = 0.12 mm^−1^
*T* = 100 K
0.43 × 0.32 × 0.13 mm

#### Data collection

Bruker SMART APEXII CCD area-detector diffractometer
Absorption correction: multi-scan (**SADABS**; Bruker, 2005 ▶) *T*_min_ = 0.949, *T*_max_ = 0.985
17515 measured reflections
3075 independent reflections
2703 reflections with *I* > 2σ(*I*)
*R*_int_ = 0.028

#### Refinement

*R*[*F* ^2^ > 2σ(*F* ^2^)] = 0.068
*wR*(*F* ^2^) = 0.173
*S* = 1.08
3075 reflections
266 parameters
45 restraints
H atoms treated by a mixture of independent and constrained refinement
Δρ_max_ = 0.66 e Å^−3^
Δρ_min_ = −0.84 e Å^−3^

### 

Data collection: *APEX2* (Bruker, 2005 ▶); cell refinement: *SAINT* (Bruker, 2005 ▶); data reduction: *SAINT*; program(s) used to solve structure: *SHELXTL* (Sheldrick, 2008 ▶); program(s) used to refine structure: *SHELXTL*; molecular graphics: *SHELXTL*; software used to prepare material for publication: *SHELXTL* and *PLATON* (Spek, 2009 ▶).

## Supplementary Material

Crystal structure: contains datablocks global, I. DOI: 10.1107/S1600536809015918/ci2792sup1.cif

Structure factors: contains datablocks I. DOI: 10.1107/S1600536809015918/ci2792Isup2.hkl

Additional supplementary materials:  crystallographic information; 3D view; checkCIF report

## Figures and Tables

**Table 1:** Hydrogen-bond geometry (Å, °)

*D*—H⋯*A*	*D*—H	H⋯*A*	*D*⋯*A*	*D*—H⋯*A*
N1—H1*N*1⋯O2^i^	0.89 (3)	2.14 (3)	3.007 (2)	165 (2)
N2—H1*N*2⋯O1^ii^	0.86 (3)	1.99 (3)	2.840 (2)	177 (3)

Symmetry codes: (i) 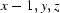; (ii) 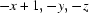.

**Fig. 1.**
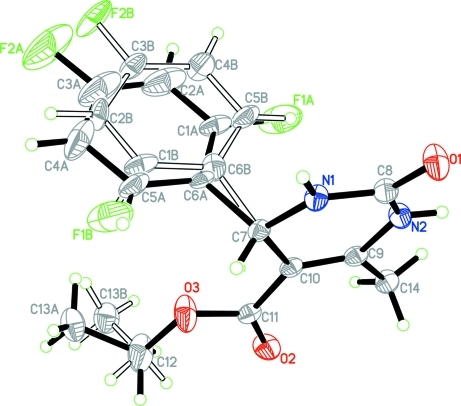
The molecular structure of the title compound, with atom labels and 50% probability displacement ellipsoids for non-H atoms. All disorder components are shown.

**Fig. 2.**
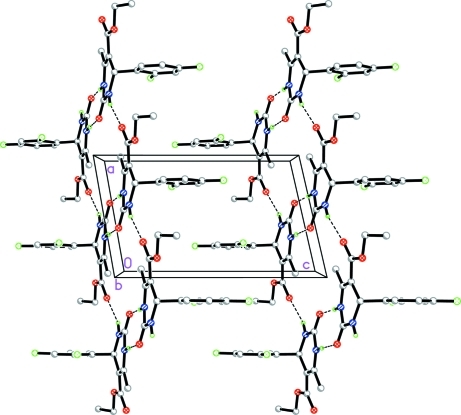
The crystal packing of the title compound, viewed down the b axis, showing chains along the [100]. Hydrogen bonds are shown as dashed lines. Only major disorder components are shown.

## supplementary crystallographic information

### Comment

Michael addition followed by aldol condensation known as the Robinson's annulation is synthetically a very useful reaction for the construction of six-membered cyclic compounds (Kalluraya and Rai, 2003). 3,4-Dihydro-pyrimidinones are compounds that have drawn wide-spread attention, due to their pharmaceutical applications (Atwal, 1990; Sadanandam *et al.*, 1992). The common synthetic routes to these compounds generally involve multi-step transformation, which are essentially based on the Biginelli condensation methodology (Steele *et al.*, 1998). These pyrimidinones are also associated with activities like calcium channel blocking (Manjula *et al.*, 2004). We synthesized the title compound by means of Robinson's annulation employing the microwave technique, and its crystal structure is reported here.

Bond lengths (Allen *et al.*, 1987) and angles in the title molecule (Fig. 1) are within normal ranges. The dihydropyrimidinone ring adopts a flattened boat conformation, with puckering parameters Q = 0.170 (2) Å, Θ = 97.4 (7)° and φ = 254.4 (7)°.

The difluorophenyl group is disordered over two positions with occupancies of 0.544 (3) and 0.456 (3). The caboxylate methyl group is also disordered over two positions with occupancies of 0.580 (8) and 0.420 (8).

In the crystal structure, the molecules are linked into centrosymmetric dimers by means of paired N—H···O hydrogen bonds (Table 1). The dimers are linked into a chain along the [100] again by N—H···O hydrogen bonds (Fig. 2).

### Experimental

A mixture of 2,4-difluoro benzaldehyde (0.01 mol), ethyl acetoacetate (0.015 mol), thiourea (0.01 mol) and conc. H_2_SO_4_ (2 drops) in absolute alcohol (10 ml) taken in a beaker (100 ml) was zapped inside a microwave oven for 3 min at 160 Watt (i.e. 25% MW power). The reaction mixture was then allowed to stand at room temperature and the product formed was filtered, washed with ethanol followed by water and dried. Further purification was done by recrystallisation from ethanol. Single crystals suitable for X-ray analysis were obtained from ethanol by slow evaporation.

### Refinement

Atoms H1N1 and H1N2 were located in a difference Fourier map and refined freely. The remaining H atoms were positioned geometrically and refined using a riding model, with C-H = 0.93–0.98 Å and U_iso_(H) = 1.2-1.5 U_eq_(C). A rotating-group model was applied for the methyl group. The difluorophenyl group is disordered over two positions with occupancies of 0.544 (3) and 0.456 (3). The caboxylate methyl group is also disordered over two positions with occupancies of 0.580 (8) and 0.420 (8). For the disordered difluorophenyl group, the same U^ij^ parameters were used for atom pairs F1A/F1B, C1A/C1B, and C5A/C5B, and all disordered atoms were subjected to a rigid bond restraint.

### Crystal data

**Table:**

C_14_H_14_F_2_N_2_O_3_	*Z* = 2
*M**_r_* = 296.27	*F*(000) = 308
Triclinic, *P*1	*D*_x_ = 1.466 Mg m^−^^3^
Hall symbol: -P 1	Mo *K*α radiation, λ = 0.71073 Å
*a* = 7.5176 (1) Å	Cell parameters from 7927 reflections
*b* = 8.0483 (1) Å	θ = 2.7–34.4°
*c* = 11.9323 (2) Å	µ = 0.12 mm^−^^1^
α = 90.147 (1)°	*T* = 100 K
β = 100.839 (1)°	Plate, colourless
γ = 108.421 (1)°	0.43 × 0.32 × 0.13 mm
*V* = 671.25 (2) Å^3^	

### Data collection

**Table:**

Bruker SMART APEXII CCD area-detector diffractometer	3075 independent reflections
Radiation source: fine-focus sealed tube	2703 reflections with *I* > 2σ(*I*)
graphite	*R*_int_ = 0.028
φ and ω scans	θ_max_ = 27.5°, θ_min_ = 2.7°
Absorption correction: multi-scan (*SADABS*; Bruker, 2005)	*h* = −9→9
*T*_min_ = 0.949, *T*_max_ = 0.985	*k* = −10→10
17515 measured reflections	*l* = −15→15

### Refinement

**Table:**

Refinement on *F*^2^	Primary atom site location: structure-invariant direct methods
Least-squares matrix: full	Secondary atom site location: difference Fourier map
*R*[*F*^2^ > 2σ(*F*^2^)] = 0.068	Hydrogen site location: inferred from neighbouring sites
*wR*(*F*^2^) = 0.173	H atoms treated by a mixture of independent and constrained refinement
*S* = 1.08	*w* = 1/[σ^2^(*F*_o_^2^) + (0.0607*P*)^2^ + 0.8775*P*] where *P* = (*F*_o_^2^ + 2*F*_c_^2^)/3
3075 reflections	(Δ/σ)_max_ = 0.001
266 parameters	Δρ_max_ = 0.66 e Å^−^^3^
45 restraints	Δρ_min_ = −0.84 e Å^−^^3^

### Special details

**Table:**

Experimental. The crystal was placed in the cold stream of an Oxford Cyrosystems Cobra open-flow nitrogen cryostat (Cosier & Glazer, 1986) operating at 100.0 (1)K.
Geometry. All esds (except the esd in the dihedral angle between two l.s. planes) are estimated using the full covariance matrix. The cell esds are taken into account individually in the estimation of esds in distances, angles and torsion angles; correlations between esds in cell parameters are only used when they are defined by crystal symmetry. An approximate (isotropic) treatment of cell esds is used for estimating esds involving l.s. planes.
Refinement. Refinement of F^2^ against ALL reflections. The weighted R-factor wR and goodness of fit S are based on F^2^, conventional R-factors R are based on F, with F set to zero for negative F^2^. The threshold expression of F^2^ > 2sigma(F^2^) is used only for calculating R-factors(gt) etc. and is not relevant to the choice of reflections for refinement. R-factors based on F^2^ are statistically about twice as large as those based on F, and R- factors based on ALL data will be even larger.

### Fractional atomic coordinates and isotropic or equivalent isotropic displacement parameters (Å^2^)

**Table:**

	*x*	*y*	*z*	*U*_iso_*/*U*_eq_	Occ. (<1)
F1A	0.7550 (6)	0.3028 (5)	0.3410 (3)	0.0598 (8)	0.544 (3)
F1B	0.8268 (7)	0.8571 (6)	0.2813 (4)	0.0598 (8)	0.456 (3)
O1	0.3876 (2)	0.1588 (2)	0.01956 (16)	0.0352 (4)	
O2	1.2723 (2)	0.5461 (2)	0.14780 (15)	0.0298 (4)	
O3	1.1456 (2)	0.7375 (2)	0.20789 (18)	0.0428 (5)	
N1	0.5906 (2)	0.4172 (2)	0.10712 (14)	0.0207 (4)	
N2	0.7014 (2)	0.1862 (2)	0.07956 (16)	0.0234 (4)	
F2A	0.7967 (7)	0.7696 (9)	0.6102 (3)	0.0959 (19)	0.544 (3)
C1A	0.7750 (7)	0.4770 (8)	0.3704 (4)	0.0279 (8)	0.544 (3)
C2A	0.7792 (9)	0.5355 (13)	0.4811 (5)	0.0557 (19)	0.544 (3)
H2AA	0.7719	0.4586	0.5394	0.067*	0.544 (3)
C3A	0.7941 (12)	0.7054 (15)	0.5035 (6)	0.061 (2)	0.544 (3)
C4A	0.8048 (8)	0.8278 (10)	0.4216 (5)	0.0520 (16)	0.544 (3)
H4AA	0.8131	0.9433	0.4381	0.062*	0.544 (3)
C5A	0.8022 (7)	0.7640 (8)	0.3129 (5)	0.0332 (10)	0.544 (3)
H5AA	0.8107	0.8423	0.2554	0.040*	0.544 (3)
C6A	0.788 (3)	0.5949 (12)	0.2835 (7)	0.0180 (17)	0.544 (3)
F2B	0.7660 (6)	0.6163 (6)	0.6323 (2)	0.0479 (11)	0.456 (3)
C1B	0.8051 (8)	0.7153 (9)	0.3476 (5)	0.0279 (8)	0.456 (3)
C2B	0.8046 (11)	0.7414 (8)	0.4604 (6)	0.0255 (14)	0.456 (3)
H2BA	0.8265	0.8519	0.4945	0.031*	0.456 (3)
C3B	0.7694 (10)	0.5921 (9)	0.5204 (5)	0.0275 (13)	0.456 (3)
C4B	0.7384 (11)	0.4271 (8)	0.4734 (5)	0.0345 (14)	0.456 (3)
H4BA	0.7125	0.3326	0.5193	0.041*	0.456 (3)
C5B	0.7443 (9)	0.3954 (11)	0.3573 (5)	0.0332 (10)	0.456 (3)
H5BA	0.7301	0.2853	0.3256	0.040*	0.456 (3)
C6B	0.774 (3)	0.5502 (15)	0.2934 (11)	0.025 (3)	0.456 (3)
C7	0.7793 (3)	0.5371 (3)	0.16198 (16)	0.0182 (4)	
H7A	0.7995	0.6433	0.1195	0.022*	0.580 (8)
H7B	0.7985	0.6385	0.1178	0.022*	0.420 (8)
C8	0.5505 (3)	0.2516 (3)	0.06653 (18)	0.0234 (4)	
C9	0.8906 (3)	0.2904 (3)	0.11002 (16)	0.0197 (4)	
C10	0.9344 (3)	0.4608 (3)	0.14452 (15)	0.0179 (4)	
C11	1.1330 (3)	0.5786 (3)	0.16561 (16)	0.0198 (4)	
C12	1.3361 (4)	0.8646 (4)	0.2385 (3)	0.0541 (9)	
H12A	1.3717	0.9251	0.1718	0.065*	0.580 (8)
H12B	1.4283	0.8057	0.2670	0.065*	0.580 (8)
H12C	1.4223	0.8259	0.2019	0.065*	0.420 (8)
H12D	1.3332	0.9763	0.2093	0.065*	0.420 (8)
C13A	1.3350 (6)	0.9926 (6)	0.3285 (4)	0.0367 (13)	0.580 (8)
H13A	1.4629	1.0700	0.3559	0.055*	0.580 (8)
H13B	1.2866	0.9305	0.3908	0.055*	0.580 (8)
H13C	1.2546	1.0599	0.2970	0.055*	0.580 (8)
C13B	1.4024 (9)	0.8870 (8)	0.3480 (5)	0.0355 (17)	0.420 (8)
H13D	1.4307	0.7841	0.3750	0.053*	0.420 (8)
H13E	1.3085	0.9070	0.3861	0.053*	0.420 (8)
H13F	1.5170	0.9866	0.3640	0.053*	0.420 (8)
C14	1.0280 (3)	0.1943 (3)	0.09888 (19)	0.0242 (4)	
H14A	1.1389	0.2365	0.1591	0.036*	
H14B	1.0657	0.2138	0.0262	0.036*	
H14C	0.9676	0.0710	0.1042	0.036*	
H1N1	0.493 (4)	0.458 (4)	0.105 (2)	0.034 (7)*	
H1N2	0.674 (4)	0.081 (4)	0.052 (2)	0.035 (7)*	

### Atomic displacement parameters (Å^2^)

**Table:**

	*U*^11^	*U*^22^	*U*^33^	*U*^12^	*U*^13^	*U*^23^
F1A	0.078 (2)	0.0668 (19)	0.0584 (16)	0.0393 (16)	0.0423 (15)	0.0248 (14)
F1B	0.078 (2)	0.0668 (19)	0.0584 (16)	0.0393 (16)	0.0423 (15)	0.0248 (14)
O1	0.0139 (7)	0.0315 (8)	0.0586 (11)	0.0057 (6)	0.0059 (7)	−0.0174 (8)
O2	0.0166 (7)	0.0307 (8)	0.0447 (9)	0.0085 (6)	0.0107 (6)	0.0018 (7)
O3	0.0160 (8)	0.0346 (9)	0.0721 (13)	−0.0006 (7)	0.0104 (8)	−0.0262 (9)
N1	0.0141 (8)	0.0243 (9)	0.0253 (8)	0.0088 (7)	0.0032 (6)	−0.0036 (7)
N2	0.0160 (8)	0.0206 (8)	0.0350 (10)	0.0063 (7)	0.0081 (7)	−0.0045 (7)
F2A	0.081 (3)	0.165 (5)	0.0359 (19)	0.034 (3)	0.0086 (18)	−0.043 (3)
C1A	0.0253 (17)	0.047 (2)	0.0141 (15)	0.0144 (16)	0.0068 (12)	0.0079 (16)
C2A	0.039 (3)	0.102 (6)	0.023 (3)	0.018 (4)	0.008 (2)	0.002 (4)
C3A	0.039 (3)	0.112 (7)	0.026 (4)	0.018 (5)	0.003 (3)	−0.023 (4)
C4A	0.030 (3)	0.071 (4)	0.049 (3)	0.012 (3)	0.002 (2)	−0.039 (3)
C5A	0.0261 (18)	0.051 (3)	0.0162 (16)	0.0024 (17)	0.0063 (13)	−0.0106 (19)
C6A	0.010 (3)	0.031 (5)	0.012 (2)	0.004 (4)	0.0039 (16)	−0.003 (2)
F2B	0.079 (3)	0.073 (3)	0.0113 (13)	0.051 (2)	0.0125 (14)	0.0029 (15)
C1B	0.0253 (17)	0.047 (2)	0.0141 (15)	0.0144 (16)	0.0068 (12)	0.0079 (16)
C2B	0.040 (3)	0.017 (2)	0.025 (3)	0.019 (2)	0.002 (3)	−0.002 (2)
C3B	0.041 (3)	0.036 (3)	0.015 (3)	0.023 (3)	0.008 (2)	0.005 (2)
C4B	0.058 (4)	0.028 (3)	0.028 (3)	0.023 (3)	0.016 (3)	0.011 (2)
C5B	0.0261 (18)	0.051 (3)	0.0162 (16)	0.0024 (17)	0.0063 (13)	−0.0106 (19)
C6B	0.016 (4)	0.025 (5)	0.036 (5)	0.008 (5)	0.005 (3)	−0.005 (3)
C7	0.0150 (9)	0.0218 (9)	0.0189 (9)	0.0073 (7)	0.0039 (7)	−0.0009 (7)
C8	0.0159 (9)	0.0252 (10)	0.0304 (10)	0.0067 (8)	0.0080 (8)	−0.0038 (8)
C9	0.0156 (9)	0.0262 (10)	0.0196 (9)	0.0084 (8)	0.0062 (7)	0.0020 (7)
C10	0.0144 (9)	0.0250 (10)	0.0159 (8)	0.0081 (7)	0.0043 (7)	0.0012 (7)
C11	0.0173 (9)	0.0269 (10)	0.0162 (8)	0.0082 (8)	0.0041 (7)	0.0013 (7)
C12	0.0205 (12)	0.0508 (17)	0.078 (2)	−0.0088 (11)	0.0148 (13)	−0.0355 (15)
C13A	0.033 (2)	0.028 (2)	0.042 (2)	0.0054 (17)	−0.0014 (18)	−0.0057 (17)
C13B	0.032 (3)	0.035 (3)	0.034 (3)	0.007 (2)	−0.001 (2)	−0.006 (2)
C14	0.0198 (10)	0.0261 (10)	0.0308 (11)	0.0112 (8)	0.0085 (8)	0.0002 (8)

### Geometric parameters (Å, °)

**Table:**

F1A—C1A	1.398 (6)	C3B—C4B	1.373 (8)
F1B—C1B	1.375 (7)	C4B—C5B	1.418 (8)
O1—C8	1.238 (3)	C4B—H4BA	0.93
O2—C11	1.211 (2)	C5B—C6B	1.441 (12)
O3—C11	1.342 (3)	C5B—H5BA	0.93
O3—C12	1.451 (3)	C6B—C7	1.579 (12)
N1—C8	1.337 (3)	C7—C10	1.524 (3)
N1—C7	1.468 (2)	C7—H7A	0.98
N1—H1N1	0.89 (3)	C7—H7B	0.96
N2—C8	1.379 (3)	C9—C10	1.349 (3)
N2—C9	1.382 (3)	C9—C14	1.496 (3)
N2—H1N2	0.86 (3)	C10—C11	1.467 (3)
F2A—C3A	1.367 (7)	C12—C13B	1.299 (7)
C1A—C2A	1.392 (8)	C12—C13A	1.490 (5)
C1A—C6A	1.404 (9)	C12—H12A	0.97
C2A—C3A	1.358 (11)	C12—H12B	0.97
C2A—H2AA	0.93	C12—H12C	0.97
C3A—C4A	1.384 (11)	C12—H12D	0.97
C4A—C5A	1.388 (7)	C13A—H12D	1.4259
C4A—H4AA	0.93	C13A—H13A	0.96
C5A—C6A	1.371 (11)	C13A—H13B	0.96
C5A—H5AA	0.93	C13A—H13C	0.96
C6A—C7	1.505 (8)	C13B—H13D	0.96
F2B—C3B	1.355 (6)	C13B—H13E	0.96
C1B—C2B	1.362 (8)	C13B—H13F	0.96
C1B—C6B	1.409 (12)	C14—H14A	0.96
C2B—C3B	1.378 (7)	C14—H14B	0.96
C2B—H2BA	0.93	C14—H14C	0.96
			
C11—O3—C12	116.68 (18)	C10—C7—H7B	104.8
C8—N1—C7	126.65 (17)	C6B—C7—H7B	121.4
C8—N1—H1N1	117.3 (18)	O1—C8—N1	123.06 (19)
C7—N1—H1N1	115.9 (18)	O1—C8—N2	120.41 (19)
C8—N2—C9	123.28 (18)	N1—C8—N2	116.53 (18)
C8—N2—H1N2	115 (2)	C10—C9—N2	119.93 (18)
C9—N2—H1N2	120 (2)	C10—C9—C14	126.95 (18)
C2A—C1A—F1A	122.6 (6)	N2—C9—C14	113.12 (17)
C2A—C1A—C6A	119.6 (7)	C9—C10—C11	120.89 (17)
F1A—C1A—C6A	117.9 (5)	C9—C10—C7	121.08 (17)
C3A—C2A—C1A	119.6 (6)	C11—C10—C7	118.04 (17)
C3A—C2A—H2AA	120.2	O2—C11—O3	121.61 (18)
C1A—C2A—H2AA	120.2	O2—C11—C10	127.53 (19)
C2A—C3A—F2A	122.2 (8)	O3—C11—C10	110.85 (16)
C2A—C3A—C4A	123.8 (6)	C13B—C12—O3	113.2 (4)
F2A—C3A—C4A	114.0 (8)	C13B—C12—C13A	46.9 (3)
C3A—C4A—C5A	114.5 (6)	O3—C12—C13A	108.3 (3)
C3A—C4A—H4AA	122.7	C13B—C12—H12A	135.7
C5A—C4A—H4AA	122.7	O3—C12—H12A	110.0
C6A—C5A—C4A	125.2 (7)	C13A—C12—H12A	110.0
C6A—C5A—H5AA	117.4	C13B—C12—H12B	64.7
C4A—C5A—H5AA	117.4	O3—C12—H12B	110.0
C5A—C6A—C1A	117.2 (6)	C13A—C12—H12B	110.0
C5A—C6A—C7	121.1 (7)	H12A—C12—H12B	108.4
C1A—C6A—C7	121.6 (7)	C13B—C12—H12C	108.9
C2B—C1B—F1B	118.1 (6)	O3—C12—H12C	108.9
C2B—C1B—C6B	124.0 (7)	C13A—C12—H12C	141.9
F1B—C1B—C6B	117.9 (6)	H12A—C12—H12C	63.9
C1B—C2B—C3B	115.2 (5)	H12B—C12—H12C	48.0
C1B—C2B—H2BA	122.4	C13B—C12—H12D	109.0
C3B—C2B—H2BA	122.4	O3—C12—H12D	109.0
F2B—C3B—C4B	120.2 (5)	C13A—C12—H12D	67.0
F2B—C3B—C2B	115.7 (6)	H12A—C12—H12D	46.1
C4B—C3B—C2B	124.1 (5)	H12B—C12—H12D	139.4
C3B—C4B—C5B	122.4 (6)	H12C—C12—H12D	107.8
C3B—C4B—H4BA	118.8	C12—C13A—H13A	109.5
C5B—C4B—H4BA	118.8	H12D—C13A—H13A	100.8
C4B—C5B—C6B	113.6 (8)	C12—C13A—H13B	109.5
C4B—C5B—H5BA	123.2	H12D—C13A—H13B	143.5
C6B—C5B—H5BA	123.2	C12—C13A—H13C	109.5
C1B—C6B—C5B	120.6 (9)	H12D—C13A—H13C	77.8
C1B—C6B—C7	119.2 (7)	C12—C13B—H13D	109.5
C5B—C6B—C7	120.1 (8)	C12—C13B—H13E	109.5
N1—C7—C6A	113.1 (6)	H13D—C13B—H13E	109.5
N1—C7—C10	109.82 (15)	C12—C13B—H13F	109.5
C6A—C7—C10	115.3 (7)	H13D—C13B—H13F	109.5
N1—C7—C6B	105.6 (8)	H13E—C13B—H13F	109.5
C10—C7—C6B	110.0 (8)	C9—C14—H14A	109.5
N1—C7—H7A	105.9	C9—C14—H14B	109.5
C6A—C7—H7A	105.9	H14A—C14—H14B	109.5
C10—C7—H7A	105.9	C9—C14—H14C	109.5
C6B—C7—H7A	119.3	H14A—C14—H14C	109.5
N1—C7—H7B	104.8	H14B—C14—H14C	109.5
C6A—C7—H7B	108.1		
			
F1A—C1A—C2A—C3A	−178.4 (6)	C5A—C6A—C7—C6B	−168 (8)
C6A—C1A—C2A—C3A	0.7 (12)	C1A—C6A—C7—C6B	10 (5)
C1A—C2A—C3A—F2A	179.2 (6)	C1B—C6B—C7—N1	−120.8 (15)
C1A—C2A—C3A—C4A	0.3 (12)	C5B—C6B—C7—N1	60.9 (18)
C2A—C3A—C4A—C5A	−1.0 (11)	C1B—C6B—C7—C6A	5(5)
F2A—C3A—C4A—C5A	−180.0 (5)	C5B—C6B—C7—C6A	−173 (8)
C3A—C4A—C5A—C6A	0.8 (12)	C1B—C6B—C7—C10	120.7 (15)
C4A—C5A—C6A—C1A	0.1 (18)	C5B—C6B—C7—C10	−57.6 (18)
C4A—C5A—C6A—C7	177.7 (8)	C7—N1—C8—O1	−178.9 (2)
C2A—C1A—C6A—C5A	−0.9 (17)	C7—N1—C8—N2	1.3 (3)
F1A—C1A—C6A—C5A	178.2 (9)	C9—N2—C8—O1	166.8 (2)
C2A—C1A—C6A—C7	−178.4 (9)	C9—N2—C8—N1	−13.3 (3)
F1A—C1A—C6A—C7	0.6 (17)	C8—N2—C9—C10	9.5 (3)
F1B—C1B—C2B—C3B	175.8 (6)	C8—N2—C9—C14	−169.96 (19)
C6B—C1B—C2B—C3B	−0.7 (16)	N2—C9—C10—C11	−173.48 (17)
C1B—C2B—C3B—F2B	−179.1 (5)	C14—C9—C10—C11	6.0 (3)
C1B—C2B—C3B—C4B	0.7 (12)	N2—C9—C10—C7	6.1 (3)
F2B—C3B—C4B—C5B	−178.7 (6)	C14—C9—C10—C7	−174.45 (18)
C2B—C3B—C4B—C5B	1.5 (12)	N1—C7—C10—C9	−15.5 (2)
C3B—C4B—C5B—C6B	−3.4 (14)	C6A—C7—C10—C9	113.7 (5)
C2B—C1B—C6B—C5B	−1(3)	C6B—C7—C10—C9	100.3 (6)
F1B—C1B—C6B—C5B	−178.0 (13)	N1—C7—C10—C11	164.14 (16)
C2B—C1B—C6B—C7	−179.8 (10)	C6A—C7—C10—C11	−66.7 (5)
F1B—C1B—C6B—C7	4(2)	C6B—C7—C10—C11	−80.1 (6)
C4B—C5B—C6B—C1B	3(2)	C12—O3—C11—O2	−4.1 (3)
C4B—C5B—C6B—C7	−178.4 (12)	C12—O3—C11—C10	176.8 (2)
C8—N1—C7—C6A	−118.4 (5)	C9—C10—C11—O2	6.0 (3)
C8—N1—C7—C10	12.0 (3)	C7—C10—C11—O2	−173.63 (19)
C8—N1—C7—C6B	−106.5 (7)	C9—C10—C11—O3	−174.99 (18)
C5A—C6A—C7—N1	−109.7 (12)	C7—C10—C11—O3	5.4 (2)
C1A—C6A—C7—N1	67.8 (15)	C11—O3—C12—C13B	−104.0 (4)
C5A—C6A—C7—C10	122.8 (12)	C11—O3—C12—C13A	−154.2 (3)
C1A—C6A—C7—C10	−59.7 (14)		

### Hydrogen-bond geometry (Å, °)

**Table:**

*D*—H···*A*	*D*—H	H···*A*	*D*···*A*	*D*—H···*A*
N1—H1N1···O2^i^	0.89 (3)	2.14 (3)	3.007 (2)	165 (2)
N2—H1N2···O1^ii^	0.86 (3)	1.99 (3)	2.840 (2)	177 (3)

Symmetry codes: (i) *x*−1, *y*, *z*; (ii) −*x*+1, −*y*, −*z*.
